# The rubral wing and its connectome

**DOI:** 10.1016/j.nicl.2025.103849

**Published:** 2025-07-20

**Authors:** Volker A. Coenen, Alexander Rau, Horst Urbach, Bastian E.A. Sajonz, Marco Reisert

**Affiliations:** a Department of Stereotactic and Functional Neurosurgery, Medical Center of Freiburg University, Freiburg, Germany; b Medical Faculty of Freiburg University, Germany; c Center for Deep Brain Stimulation, Medical Center of Freiburg University, Germany; d Department of Neuroradiology, Medical Center – University of Freiburg, Germany; e Department of Diagnostic and Interventional Radiology, Medical Physics, Medical Center – University of Freiburg, Germany

**Keywords:** Rubral wing, Tremor, Deep brain stimulation, FGATIR sequence, FLAWS sequence, High intensity focused ultrasound, Stereotactic radiosurgery

## Abstract

**Purpose:**

Imaging developments optimizing stereotactic surgery for tremor have yielded white matter attenuating sequences (FGATIR/FLAWS) showing a targetable hypointensity in the subthalamic region (rubral wing, RW). RW has been reported to coincide with yet to be defined portions of the dentato-rubro-thalamic tract (DRT). Its discernibility on the single subject level might be hampered by low signal-to-noise ratio (SNR), potentially compromising with surgical outcomes. We here set out to investigate RW as a target structure for tremor surgery by further characterizing it volumetrically and with tractography.

**Methods:**

We performed manual delineations of RW on FLAWS sequences (n = 77, 3 raters) and warped results into MNI 152. We performed tractographic analyses of DRT (human connectome project body, n = 1000), using the red nuclei and RW as waypoints. Further, we investigated fiber tightness peaks of crossed (DRTx) and uncrossed DRT portions (DRTu) along their z-axis.

**Results:**

Identification of RW was possible in all subjects. DICE coefficient for volumetric comparison was rather low with 0.54. Euclidean distance of the RW centers of gravity (COG) was <2 mm. RW defines DRTx and optimally routes fibers to the precentral gyrus (PCG). A tightness peak for fibers descending from PCG (DRTx only) was found to coincide with RW.

**Conclusions:**

RW potentially represents a valid candidate target for tremor surgery and is part of the post-crossing portion of the DRTx. COG of RW can be determined with good accuracy, but full volumetric appreciation is difficult on the single subject level (SNR), potentially necessitating additional imaging for surgical targeting.

## Highlights

• The Rubral Wing is a precise anatomical waypoint for the crossed dentatorubrothalamic tract (DRTx).
• RW has strong connectivity to the precentral gyrus.
• FGATIR/FLAWS MRI allows subject-level visualization of RW  though single-subject volumetry is limited by low SNR.
• RW rendition with FGATIR/FLAWS may improve DRT targeting in tremor surgery without relying on DTI.
• RW targeting might offer clinical relevance for DBS and SLS planning including MRgFUS and radiosurgery.

## 1 Introduction

Surgical techniques for stereotactic approaches like deep brain stimulation (DBS) or stereotactic lesion surgery (SLS) for pharmaco-therapy refractory tremor syndromes have shown a dramatic development especially in the realm of imaging technologies for sophisticated targeting (Bot et al., 2022, Chan et al., 2023, Coenen and Reisert, 2021, Feltrin et al., 2022, Middlebrooks et al., 2020, Middlebrooks et al., 2021a, Neudorfer et al., 2022). Besides the traditional approaches for target region identification, diffusion weighted magnetic resonance imaging (DWI)-based tractography allows for a relatively accurate definition already during planning. For instance, this facilitated the identification of the dentato-rubro-thalamic tract (DRT) as a potentially common target structure for tremor surgery (Coenen et al., 2020, Middlebrooks et al., 2021b, Middlebrooks et al., 2023). As the usage of this DWI-based treatment planning is not as straightforward as simply relying on conventional qualitative MRI gray images, some authors criticize these secondarily derived and coloured target renditions and question their utilization (Hariz and Blomsted, 2023). More recent additions to MRI sequence development rely on techniques with advanced capability to delineate specific brain regions usable for direct planning of stereotactic procedures. The results of such sequence development are gray images which have been preprocessed on the MRI console and are ready for immediate further use; Already in 2009 Sudhyadhom and colleagues described the FGATIR (fast gray matter acquisition T1 inversion recovery) MRI sequence for a more precise identification of subcortical white matter structures in conjunction with a gray matter environment (Sudhyadhom et al., 2009). Further sequences have been developed such as the white matter nulled (WMN) MPRAGE (magnetization prepared rapid gradient echo) (Saranathan et al., 2015) and the FLAWS (fluid and white matter suppression) sequences (Chen et al., 2018) that visualize small white matter volumes in relation to gray matter by decreasing the white matter signal, and produce a contrast relatively similar to FGATIR.

**Fig. 1:**
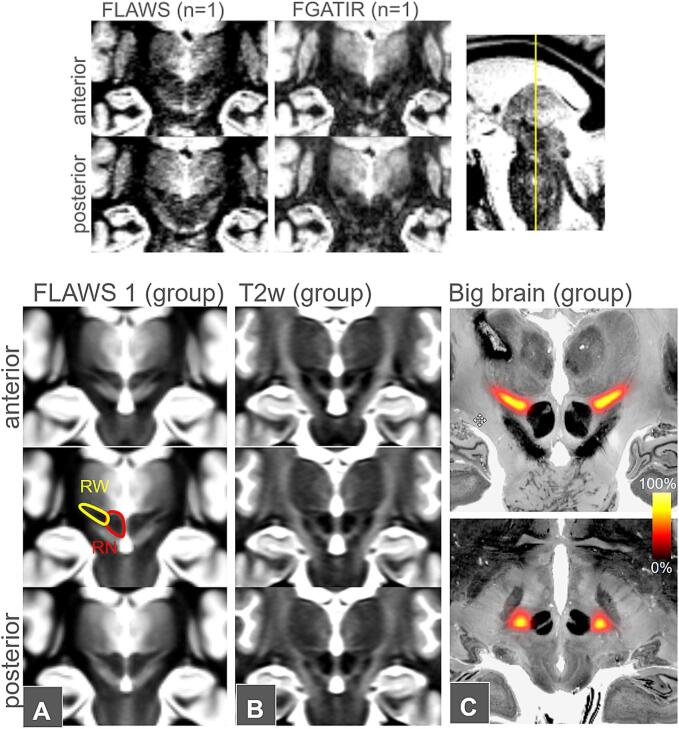
Single subject / group level comparisons of RW depiction with FLAWS and FGATIR sequences. Upper panel, typical random examples of side-by-side comparison of FLAWS and FGATIR-depiction of the rubral wing. The images show the difficulty of determining the RW at given SNR. The contrasts of FLAWS and FGATIR are similar with potentially a bit smoother appearance in FGATIR. Lower panel, A,B group averages over n=77 FLAWS and T2w contrasts in MNI space. Red outline shows the red nucleus (RN) and the rubral wing (RW). On the average contrast the RW can be delineated very well. C, RW delineation results averaged over three raters (n=77 subjects, FLAWS) overlaid on Big Brain in a coronal (upper image) and axial slice (lower image).

The utilization of such advanced MRI sequences to identify a subthalamic hypointensity as a target region for stereotactic tremor surgery was almost simultaneously described by the Amsterdam and the Berlin groups (Bot et al., 2022, Neudorfer et al., 2022). Ever since, the FGATIR hypointensity has not only been used for DBS but also for MR-guided transcranial focused ultrasound (MRgFUS) (Shah et al., 2020) and stereotactic radiosurgery (SRS) targeting (Ankrah et al., 2023). Based on its visual characteristics in coronal images, Bot & Schuurman described the subthalamic hypointense tremor target structure based on its shape as the *rubral wing* (RW) (Fig. 1), a flap-like structure, based on the red nucleus extending quasi-horizontally under the ventrolateral thalamus (Bot et al., 2022). Bot et al. used tractographic techniques to relate the RW with deterministic DRT renditions (Bot et al., 2022). In a somewhat more elaborate approach, Neudorfer et al. (Neudorfer et al., 2022) utilized stereotactic and white matter atlases to investigate the relation of the RW with the DRT. However, beyond a pure correlation of RW and DRT, there might be a clinical need for an unambiguous identification of a crossed (DRTx) and uncrossed (DRTu) portion of the DRT. Especially using a DWI tractographic depiction, the distinct sub-portions have been related to side effects of DBS and been extrapolated to SLS (Sajonz et al. 2022, 2024). Such a distinct depiction, however, might not easily be achievable on the single case basis with every clinical (deterministic) DWI tracking approach at hand (Coenen et al. 2021).

Neudorfer and Bot provided some evidence on the relation of the RW and the terminal (or horizontal) part of the DRT, however, lacking information about critical aspects which define the questions addressed in this work: 1. How decisive is the RW’s definition in a FLAWS or FGATIR sequence, justifying its use on a single case basis for stereotactic targeting? 2. Do other potentially far-reaching RW connectivities exist which have to be considered upon using the structure as target for stereotactic approaches? 3. Does the RW define the terminal portions of the uncrossed or crossed DRT or both?

To address these questions, we performed in-depth anatomical and connectomic analyses of the RW by delineating the structure in FLAWS (very similar to FGATIR) sequences, typically used in-house. For this, we delineated the RW on a single center subject sample, warped this definition to a normalized space (MNI) and performed tractographic studies in a large number of subjects (n = 1000, Human Connectome Project). Analyses were supplemented by cortical fiber penetration studies and by comparisons with stereotactically placed DBS electrodes from a clinical cohort of patients with successful DBS for tremor. This work tries to extend our own previous research. By utilizing DTI tractographic and volumetric approaches, it strives to better anatomically characterize a target region − the RW − that is already in use by specialized groups who perform tremor surgery and is directly visible in FGATIR/FLAWS sequences.

## 2 Methods & material

### 2.1 Ethics

We retrospectively identified patients from our tertiary referral center clinical registry who received in-house FLAWS or FGATIR MRI sequences (institutional review board of the University of Freiburg, no. 400/20 and 22-0001) between 2018 and 2023. Additionally, patients who had previously undergone DBS tremor surgery at our institution were selected for the participation in this study and included if they had given written informed consent for participation (institutional review board of the University of Freiburg, no. 21-1274 & no. 207/15).

### 2.2 Rubral wing delineation

We retrospectively queried our internal clinical imaging archive for FLAWS (or FGATIR) protocols in the date range from 2018 to 2023. Overall, we identified 77 imaging studies with appropriate sequences in the context of clinical imaging. All identified individuals had a conventional isotropic resolution T1-weighted MPRAGE (TR/TE/TI = 988,2300 ms/2.26 ms/988 ms), too. Patients with a neurologic condition such as stroke or hydrocephalus, a primary psychiatric disorder, or a medical condition causing cognitive difficulties were excluded. In addition, patients with movement artifacts on imaging were excluded. The FLAWS was acquired with TR/TE/TI = 5000 ms/2.43 ms/409 ms for the first inversion time following (Chen et al., 2018). We found only < 5 studies with a proper FGATIR sequence (Sudhyadhom et al., 2009), so refrained from analysing those and concentrated on the FLAWS. Fig. 1A,1B shows a comparison of FLAWS and FGATIR.

Three independent raters (MR, VAC, AR) delineated the hypointense area on the FLAWS contrast in three-dimensional reformats on a local instance of the post-processing platform NORA (https://www.nora-imaging.org). For a homogeneous process, the axial reformat was kept in ACPC orientation. Prior to labelling, raters met for a training session to agree on the precise delineation of the rubral wing as it can be estimated from the current literature (Bot et al., 2022, Neudorfer et al., 2022). After that, raters proceeded individually. To quantify the inter-rater agreements, we evaluated the DICE coefficients and the Euclidean distances of the center of gravities of the manually drawn regions.

### 2.3 Tractographic analyses of RW connectomics

To compare the RW delineations and use them subsequently for tractography, we mapped the individual space to a common group space (MNI). Therefore, we used the T1w imagery as reference and the normalization approach from (Reisert et al., 2022) based on deep learning. The delineations were warped to MNI space on a 1 mm^3^ matrix and a ‘mean’ RW was computed by simple averaging.

The diffusion MRI (dMRI) data of 1000 participants of the Human Connectome Project (3 T HCP) dataset (https://ida.loni.usc.edu/login.jsp) were employed to investigate the connectomics of the RW. These datasets feature a resolution of 1.25 mm isotropic and three diffusion shells (b = 1000, 2000, and 3000 s/mm^2^). For further details on the acquisition protocol and preprocessing, refer to (Glasser et al., 2013). Images were spatially processed using the warping fields generated by the HCP pipeline.

### 2.4 Tracking

Tractography was carried out with a global approach (Reisert et al., 2011). Whole-brain connectomes were first reconstructed from individual DWI data on the HCP corpus. ROIs, defined in atlas space using MNI coordinates or explicit masks, were then warped into participant space to extract specific bundles from the pre-computed connectomes. This independent tracking process avoids biases from predetermined starting and stopping points. Global Gibbs tractography (Reisert et al. 2011) uses a random point process to generate streamlines, optimizing their alignment with the diffusion MRI data to explain the signals. This method is noise-robust, and fiber densities reflect the measured data (Fillard et al., 2011, Reisert et al., 2011, Schumacher et al., 2018). We employed the method outlined by (Reisert et al., 2011), using the ‘dense’ preset, which adjusts parameters based on resolution and diffusion signal. This setting generates roughly 150,000 streamlines per HCP dataset. To improve reproducibility, we used an accumulation strategy: after cooling-down, the temperature was raised to 0.1 and iterated for 10^7^ courses. This process was repeated five times, resulting in a tractogram approximately five times larger, with around 800,000 streamlines per dataset. Proposed by (Schumacher et al., 2018), this strategy enhances re-test reliability. The reconstruction covered all white matter regions from the HCP parcellation, with the white matter mask slightly dilated to include the white/gray matter transition zone.

### 2.5 Bundle selection

After tractographic reconstruction, the DRT and streamlines associated with the classical DRT and rubral wing (RW-DRT) were identified using the following automated selection protocols: 1) RW-DRT: streamlines visiting a rubral wing density mask at a threshold of 0.6 were selected and either the ipsi- or contralateral superior cerebellar peduncle (SCP, defined as a sphere centered at coordinates ±7, −41, −26 with a radius of 4 mm in MNI space) was used to disentangle crossing from uncrossing streamlines. 2) The DRT itself is classically defined based on its connectivity between the precentral gyrus (PCG, defined using the Desikan-Killiany atlas (Desikan et al., 2006)) and the ipsi- and contralateral superior cerebellar peduncle (SCP) for crossing and uncrossing DRT, respectively. While the red nucleus serves sometimes as an additional waypoint, it is not completely mandatory, depending on the tractographic method. We followed two approaches here: For an understanding of the cortical projections, we left out the cortical constraint (PCG) and solely used SCP and RN for selection. Secondly, to show that the RW is a very specific waypoint for the DRT, we used solely SCP and PCG for selection, i.e. there is no intermediate on its course from the cerebellum to cortex (see below).

To aggregate participant data at the group level, streamline terminal maps were rendered onto an isotropic matrix with a 1.25 mm resolution. These density maps were then warped to MNI space using HCP-derived warping fields (onto an isotropic matrix with a 2 mm resolution) and subsequently averaged. No gray-value modulation by the determinant of the Jacobian was applied for the streamline terminal maps.

### 2.6 DRT specificity profiles

To further elucidate the RW's connection with distinct anatomical pathways of the DRT, we analyzed streamline density profiles along the MNI z-axis of the DRT which was defined by visiting PCG and SCP. We hypothesized that the RW serves as a key waypoint region for the DRT, i.e. the DRT forms a narrow bottleneck at the level of the RW. To corroborate this, we evaluated two measures for each xy slice: 1) the maximum streamline density within the slice, providing insight into the peak density distribution, and 2) the inverse diversity within the slice, quantified using the entropy and expressed as exp(−entropy). The entropy calculation involved normalizing the density of each slice to ensure a total mass of 1, enabling a standardized comparison of density profiles. In Fig. 3A,C, the DRT profiles along the z MNI coordinates are shown together with the location of the RN, the ventral intermediate nucleus (VIM) and the RW.

**Fig. 2:**
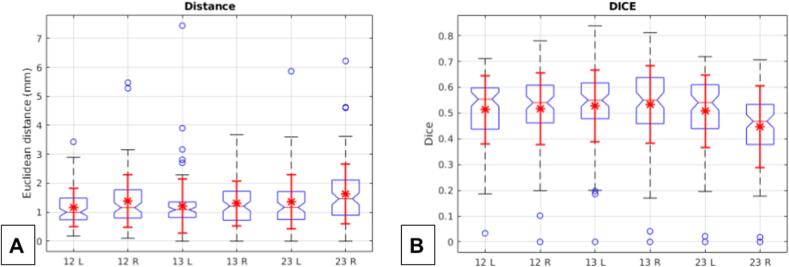
A, Whisker plots of Euclidean distances between center of gravities of the RW mask between rater 1-2, 1-3 and 2-3 for the left (L) and right (R) hemisphere. B, Corresponding whisker plots of the DICE coefficients.

**Fig. 3:**
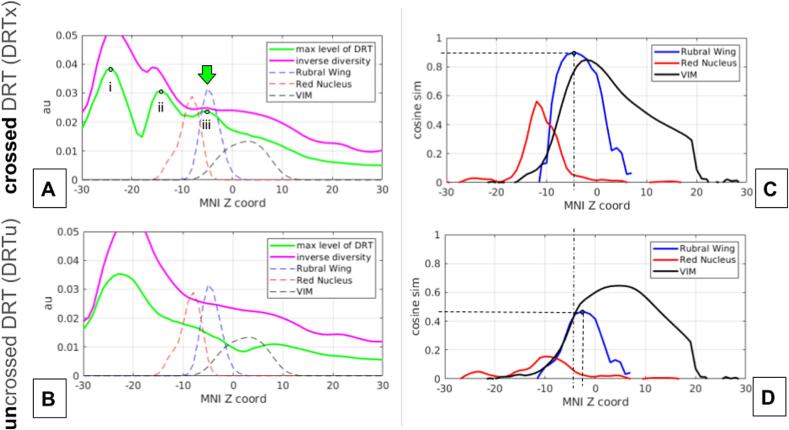
A,B: Tightness measures of the DRT (crossed/uncrossed) along MNI Z axis. The inverse diversity (negative exponential of Shannon-entropy) of the axial slice shows a peak exactly at the location of the Rubral Wing (green arrow). Peaks i-iii: i, scp; ii, cw; iii, RW. Similarly, but to a lesser extent, the maximal amplitude shows the tightness of the DRT at the level of the Rubral Wing. C,D: Cosine similarities of the DRT (crossed/uncrossed) streamline densities with the Rubral Wing/Red Nucleus/VIM. The rubral wing shows the highest similarities with DRTx (C) among the structures. Highest cosine similarity of DRTu is shown with VIM (D). Legend: scp, superior cerebellar peduncle; cw, commissure of Wernekinck (of brachium conjunctivum); RW, rubral wing.

### 2.7 DRT overlap with RW

Additionally, to measure the overlap of the relevant nuclei (RN,RW and Vim) and DRT we computed for each slice the cosine similarity of the DRT density and group averaged RW masks, i.e. the resulting number range between −1 and 1 where a value of 1 means perfect agreement between the DRT and the nuclei. Technically, we first normalized the streamline densities of the DRTu/DRTx in an Euclidean sense, and the RW group average mask likewise. Again, as above, we did this slice by slice. Then, the normalized densities are multiplied point-wise and summed up over each slice resulting in the similarity profiles visualized in Fig. 3C,D.

### 2.8 Comparison with tractographically planned thalamic / subthalamic DBS of the DRT

To investigate the agreement of potential RW targeting and established tractographic DRT planning (Coenen et al., 2021, 2020), we retrospectively analysed 35 patients, who underwent thalamic DBS for the treatment of pharmaco-refractory essential tremor or tremor from Parkinson’s disease at our center. This surgery has typically been guided by DRT tractography and has not used any RW visualization. Following the approach in (Reisert et al., 2022), we performed the retrospective imaging workup and transformed the effectively stimulated electrode contact positions into MNI space for comparison in order to investigate potential proximity to the RW and the DRT. Demographics and outcomes of this retrospective patient cohort were collected via a patient chart review. Clinical improvement − wherever available − was determined at the latest noted time point (in a 6–18 months post surgery interval, mostly at the 12 months time point). With respect to tremor clinical global impression (CGI) was used with the following grades: 1 very much improved; 2 much improved; 3, minimally improved; 4, no change; 5, minimally worse; 6, much worse; 7 very much worse. To quantify the activation of the DRTx and DRTu, we averaged the streamline density maps within the volume of activated tissue (VAT), which we computed based on individual electrode configurations. Activation threshold and tissue conductivity is chosen such that a current of 1 mA leads to an activation radius of 2 mm. To quantify the “activation” of the RW we computed the relative overlay of the VAT and the probability weighted RW mask. To explore the relationship between clinical impression (CGI score) and the activation measures, we performed univariate linear regression analyses using Python (version 3.11) with the statsmodels (version 0.14) and seaborn (version 0.12) libraries. Each activation variable was entered as an independent predictor, with CGI as the dependent variable. In Fig. 6, for each model, we report the regression slope, intercept, p-value, and coefficient of determination (R2) and 95 % confidence intervals (CI).

## 3 Results

### 3.1 Interrater-reliability of RW delineation

The manual segmentation was performed by three experienced raters by delineating the FLAWS hypointense RW structure starting in coronal images (volumetry), covering the entire discernible structure. Group-level delineation results are presented in Fig. 1 and show very good delineation of the RW structure. Interrater agreement was acceptable with a median DICE coefficient of 0.54, which would, however, not allow reproducible volumetric statistics. Additionally, correlation analysis of RW volumes between the raters did not show a significant relationship (p > 0.1). However, the distances between the center of gravities (choice of coronal image) were in a small range of mostly < 2 mm. Fig. 2 depicts corresponding whisker plots.

### 3.2 Specificity of the Rubral Wing for the crossed DRT and not uncrossed DRT

Fig. 3 shows profiles of the ipsi- (DRTu) and contralaterally (DRTx) proceeding DRT along the MNI z coordinate. Along the entire PCG seeded DRT structure we found three bottlenecks that show tightening of the fibers manifesting as peaks (i-iii) in the inverse diversity measure. Comparisons of the location of these peaks with the location of candidate structures like red nucleus, ventral intermediate nucleus and RW allow us to understand their relationship to the DRT. The peaks for the DRTx are identified as i, superior cerebellar peduncle (z = -25); ii, commissure of the superior cerebellar peduncle (z = -15, Wernekinck) and iii, most notably the rubral wing (z = -6). Indeed, the DRTu does not exhibit a peak at the level of the rubral wing and obviously not at the level of the commissure. When looking at the overlap/similarity measurements in Fig. 3 B/D, again the specificity of the RW with respect to the DRT can be noted. At around z = -6 the RW reaches the overall highest DRTx similarity of around 0.8 amongst the considered structures. A similar behaviour cannot be observed for the DRTu.

### 3.3 Cortical extent of the RW-DRT on the HCP connectome

**Fig. 4:**
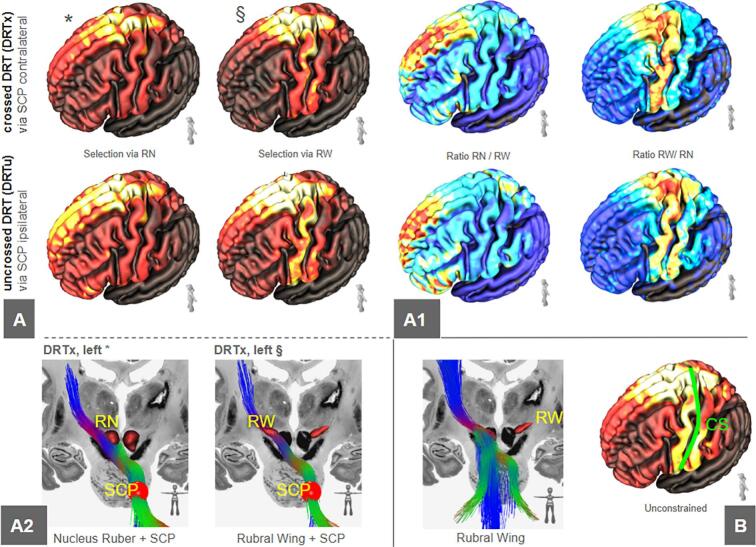
A, terminal densities of the dentato-rubro-thalamic tract connectomes  (DRTx and DRTu, respectively) constrained via red nucleus (RN) or rubral wing (RW) shown on the left. A1, shows ratios of terminal densities between the two different selection strategies. The RW selection leads to pronunciations of the precentral gyrus, while the RN selection prefers prefrontal streamlines. A2 visualizes the two selection strategies (via RN,*; via RW §) for crossed DRT (DRTx left). B, as a comparison shows a completely unconstrained selection of the streamlines via RW. Legend: RN, red nucleus; RW, rubral wing; SCP, superior cerebellar peduncle; CS, central sulcus.

The cortical densities of RW-DRT have a higher tendency to end in the PCG than the classically defined DRT. For the classical DRT which is constrained via red nucleus (RN) as waypoint, we find some more spurious density around the dorsolateral prefrontal cortex (Fig. 4).

### 3.4 Anatomical overlay correlation with DBS for tremor

Demographics and clinical outcomes: We report n = 35 patients (20 male, 15 female) with a mean age of 66.1 years (range 29.9–80.7 years). Diagnoses were: Essential tremor (28), tremor in Parkinson’s disease (2), orthostatic tremor (2), dystonic head tremor (2) and tremor-ataxia syndrome (1). Outcomes were favourable in mean with a CGI of 1.9 over all patients, 1.79 for essential tremor alone (Of note: CGI is regarded favourable from grades 1–3). Two Essential tremor patients and one Parkinson’s disease patient were graded with CGI of 4 (no change).

**Fig. 5:**
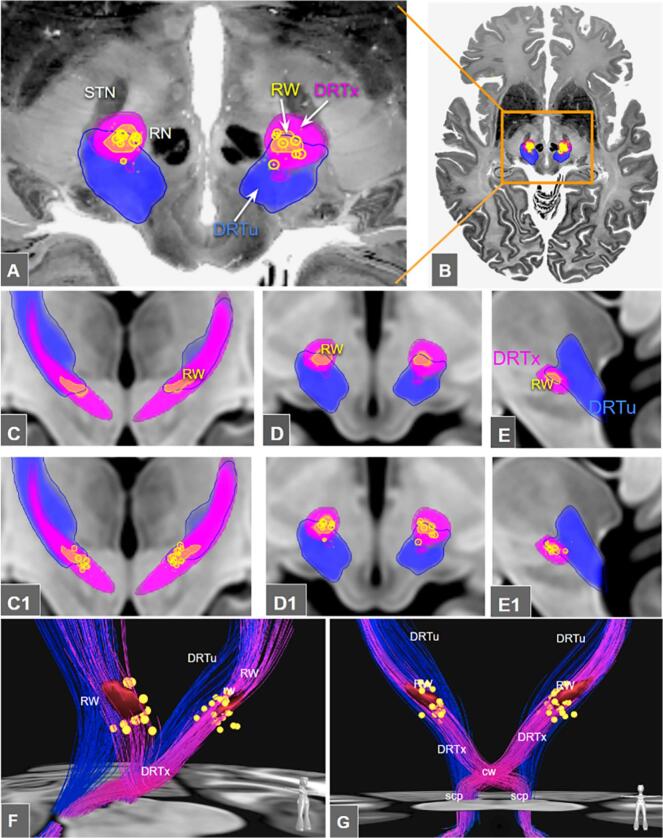
A-E, The crossed (DRTx, magenta) and uncrossed DRTs (DRTu, blue) in relationship to the rubral wing (RW, yellow) and a group of patients with tractographically planned thalamic / subthalamic DBS (electrode contacts, yellow dots) on an in-house dataset. A-B, on an axial level overlaid with the bigbrain, C-E, coronal/axial/sagittal views. C1-E1, with superimposed effective electrode contact positions from a cohort of an ET (n=28) group analysis. F-G, three-dimensional depiction. RW now red, yellow spheres represent DBS electrodes placed for tremor reduction.  Legend: scp, superior cerebellar peduncle; cw, commissure of the scp (Wernekinck); RN, red nucleus.

Fig. 5 illustrates the anatomical correlation of effective electrode contacts in a group of patients who underwent successful ventral intermediate nucleus (Vim) / DRT DBS for tremor. The electrode contacts, indeed, form a cluster around the RW further following the streamlines of DRTx, clearly avoiding DRTu fibers. The center of gravity of the cluster of contact lies at (−10.5, −18.5, −5.7) and (10.0, −17.8, −5.9) in the MNI coordinate system, respectively. On the other hand, the COG of the RW lies at (−12.2, −17.6, −4.6) and (12.0, −17.3, −5.1), i.e. slightly superior and more laterally. Measuring the minimum distances of the contacts to the RW we found the distances to be 1.14 mm + -0.95 (left) and 0.96 mm + -0.96 (right).

### 3.5 Outcome correlations

**Fig. 6:**
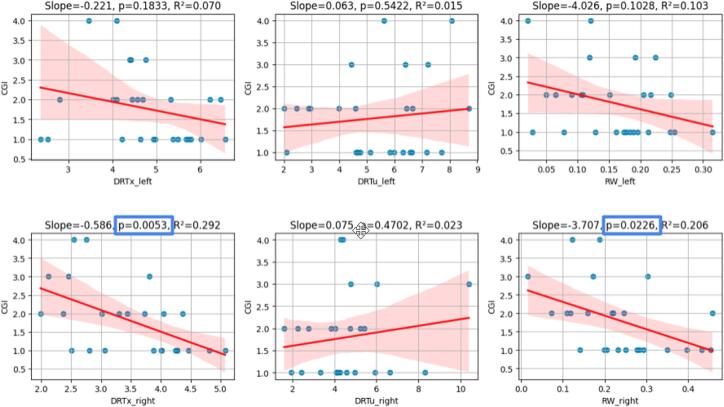
Univariate regression analysis of DRT/RW activation with clinical outcome score (CGI); n=28. Legend: RW, rubral wing; DRTx, crossed dentato-rubro-thalamic tract, DRTu, uncrossed dentato-rubro-thalamic tract.

Fig. 6 shows results of our regression analyses for a subgroup of n = 28 patients with ET implanted with DBS electrodes targeting for the Vim/DRT. Correlation is significant for the right sided electrode evaluation only and here especially for DRTx (p = 0.0053, R^2 = 0.292) and RW (p = 0.0226, R^2 = 0.206).

### 3.6 Evaluation of detailed anatomical environment

**Fig. 7:**
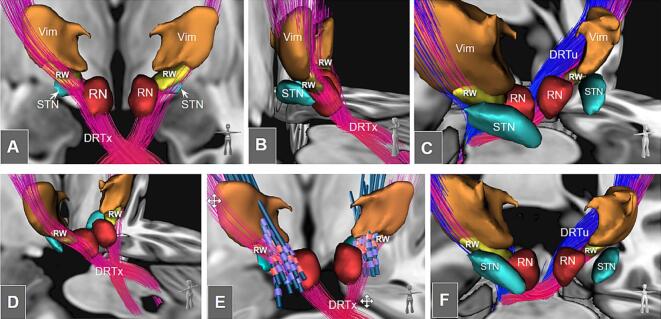
Detailed anatomical environment of RW. A-F, the additional depiction of three-dimensional structures surrounding RW and DRT explain the anatomical environment. E, essentially recapitulates Figure 5 (F-G) but now includes DBS electrode leads. Legend: DRTx, crossed DRT; DRTu, uncrossed (ipsilateral) DRT;  RW, rubral wing; STN, subthalamic nucleus;  RN, red nucleus;  ViM, ventral intermediate nucleus of thalamus.

Fig. 7 summarizes the evaluation of the exact anatomical environment of the RW. As expected, RW is located just below the Vim extending laterally from RN. DRTx (not DRTu) fibers penetrate RW.

## 4 Discussion

In this study, we set out to closely investigate the rubral wing, a typically hypointense structure in the subthalamic region that can be identified in white matter attenuation MRI sequences (FGATIR, FLAWS, WMN MPRAGE) and has recently been used as a target for tremor surgery in a multitude of settings (Bot et al., 2022, Chan et al., 2023, Middlebrooks et al., 2020, Middlebrooks et al., 2021b, Neudorfer et al., 2022). On the one hand, we investigated our in-house FLAWS sequence for its potential to allow for a volumetric rendition of RW in MNI space in a set of n = 77 subjects. Due to the relatively noisy appearance of the RW in the FLAWS contrast, a volumetric outlining of RW on an individual level is hardly possible and absolute volumes cannot be robustly determined. However, RW‘s center of gravity can be reliably determined while the anterior and posterior borders appear to be less clearly defined. Moreover, we investigated the connectomic composition of RW in a large number of additional subjects (n = 1000, HCP body). We found that RW is, with its white matter position, in a way the most specific waypoint to select the DRT. This holds true, in particular, for the crossed portion of the DRT (DRTx) and not the DRTu and RW constrained fibers are optimally routed to/from the PCG. The cortical penetrations (Fig. 4) suggest that the amount of non-DRT streamlines running through the RW is actually relatively small; no other far-reaching connections of RW seem to exist. The cortical terminals of RW-DRT are well concentrated around the precentral gyrus, which is less the case for the RN waypoint, the fibers of which extend further into the PFC. This finding is significant because it defines the RW as an exclusive penetration structure of the DRTx while not applying any DTI technology for its visualization. Conversely, RW might hold for the application as a true tractography way point for a more optimal definition of the DRT (Fig. 4, lower panel) a feature we will further follow in our future research. To our knowledge this is the first description of the RW connectome on a large number (n = 1000) of subjects in a common space.

FLAWS vs FGATIR: The initial definitions of a subthalamic hypointensity as target for tremor surgery (RW) were based on the FGATIR sequence. We do here regard the FLAWS sequence as rather similar to FGATIR and use it accordingly to investigate RW topography, volumetry and connectivity. Indeed, the first sub–acquisition in FLAWS is timed to *null (suppress) white matter* while retaining gray matter signal; it produces an image with high gray matter contrast—very similar to what FGATIR is designed to do. FGATIR also uses an inversion recovery approach that suppresses or reduces signal from surrounding tissues (especially white matter) to highlight subcortical gray matter structures. Taking this into account we think that our approach is not only valid but might be extendible to other WM attenuation sequences.

Single subject FLAWS delineation: Fig. 1 demonstrates how low SNR can be for FLAWS and FGATIR. Occasionally, this low SNR in clinical image data sets might hamper the full appreciation of RW in its full extent in the single subject. Delineation problems typically arise at its anterior and posterior border, leading to a low reproduction quality of the entire RW volume (DICE coefficient 0.54, Fig. 2B) while the mere appreciation of the overall center of the ap extent (choice of a single coronal image defining the RW’s center of gravity, COG) appears to be be achievable with acceptable accuracy (Fig. 2A). While the COG definition might be enough to somewhat guide tractographic approaches or to perform quality checks of these, we are uncertain if the data quality suffices for an entire stereotactic target definition approach in every case. This instance has the potential to lead to eccentric (too far posterior) lesions or DBS electrode positions, potentially encroaching on minimal parts of the DRTu with all the previously described and expected detrimental consequences (Sajonz et al., 2024, 2022). Fig. 5 (C-E) suggests that a minimal amount of DRTu fibers will reside in most posterior parts of RW, only a result which is further underpinned by our cosine similarity analysis (Fig. 3 C-D). In situations with a low SNR additional DTI tractographic approaches might therefore be advisable for the appreciation of an intended y-coordinate. In this context it is, however, interesting how well the entire RW volume can be depicted on group level images for FLAWS and actually also T2w sequences (Fig. 1A-B), potentially pointing towards an augmentation of single subject data with group level information.

Fiber density peaks of fibers descending out of PCG region and PCG connectivity as determinants for trueness of DRTx: We have performed a group level anatomical overlay analysis to look for fiber waypoints in the z-axis of fibers descending from PCG to the contralateral cerebellum (Fig. 3). Waypoints are identified as a culmination or tightening of fibers resulting in certain peaks along the structure. We identified three such peaks (i-iii) which represent superior cerebellar peduncle (scp, i), commissure of the scp or commissure of Wernekinck (cw, ii) and RW (iii) (Fig. 3). This analysis also reveals that DRTu does not show any further than peak one (i, scp) indicating that it does not relate to cw and more importantly not to RW. Previous work has shown that the main connectivity of effective thalamic stimulation needs to reach PCG (M1) (Akram et al., 2018, Al-Fatly et al., 2019, Coenen et al., 2021, Coenen et al., 2017, Coenen et al., 2014, Coenen et al., 2011, Fenoy and Schiess, 2017, Middlebrooks et al., 2021a, Middlebrooks et al., 2021b, Nowacki et al., 2022, Pouratian et al., 2011) strengthening the anatomical validity of our approach. During a further characterization of RW as a structure with our global tracking approach we were not able to identify any further far-reaching connectivity (Fig. 4B).

Outcome correlation: Our outcome correlation analysis (Fig. 6) partially confirms the role of RW as a valid tremor reducing structure also when targeted with DBS. Recent literature, however, shows that the typical DBS stimulation sweet spot might be located somewhat distant (typically more posterior and inferior) from a MRgFUS lesion sweet spot (Chua et al., 2025). We will not further discuss the reasons here. It would, however, not be implausible that a correlation in a DBS cohort is found with DRTx and not RW.

RW in relation to other areas: The subthalamic region shows complex and intricate fiber anatomies. Several other structures have been mentioned in the subthalamic region which are somehow related to RW most prominently the zona incerta (ZI) with the caudal zona incerta (cZI) and the pre-lemniscal radiations (PR). Especially the definition of PR is somewhat ambiguous and initially described fibers located in front of the medial lemniscus. In the original sense Hassler had conceptualized PR as being proprioceptive fibers from the medial lemniscus that enter the Vim nucleus (Hassler et al., 1979, Lévy et al., 2020). It is now, however, loosely established that the term indicates pallidothalamic as well as cerebellothalamic fibers. Accordingly, in recent work based on tractography (García-Gomar et al., 2017) the authors describe PR as a heterogeneous group of fibers that contain connections to the cerebellum, motor cortex, supplementary motor cortex, prefrontal regions, basal ganglia and some brainstem nuclei. In their work on DBS to the cZI nucleus (Plaha et al., 2008) the authors explain that the PR consists of white matter located between the medial border of the subthalamic nucleus (STN) and the lateral border of the red nucleus, with its posterior extent limited by the caudal zona incerta (cZI) and the postero-medially placed medial lemniscus. The PR is described as being located more medial and deeper than the cZI target used in their study (Plaha et al., 2008). PR pallidal territory is located further anterior, cerebellar territory more posterior. Potentially PR represents an older term from stereotaxy literature and is in itself more confusing than helping the matter. It is certainly not a sharp functional anatomical term since it does not differentiate pallidal and cerebellar territory. Gallay and Moro have in their work meticulously researched the topographical relationships in the subthalamic region in 5 cadaver brains (Gallay et al., 2008). In their work ZI is clearly a nuclear (grey matter) structure located superior (dorsal) to the STN. It is ensheathed in fibers of Forel’s field H1 (above) and H2 (below). RW as such is located above (superior or dorsal) to ZI (please note, RW is mentioned as fct (fasciculus cerebello-thalamicus) in this anatomical work) (Gallay et al., 2008). It becomes clear that ZI reaches on top of the STN into the posterior cerebellar region of the PSA where it becomes cZI. We have previously expressed in a somewhat parsimonious and “fiber-centric” view that the terms cZI, and posterior subthalamic area potentially point to cerebello-thalamic fibers (in fact the DRT) (Coenen et al., 2014) and as such analogous to RW. Our work, however, was not intended to solve nomenclatural ambiguities in the region.

## 5 Limitations

This work has some limitations that need to be addressed: RW was initially defined in the FGATIR-sequence (Bot et al., 2022, Neudorfer et al., 2022) but we here almost exclusively utilized the FLAWS sequence (Chen et al., 2018). As we have pointed out, FGATIR and FLAWS both attenuate the WM signal and by that visualize a mix of grey and white matter especially in the depth of the brain. Fig. 1 nicely shows that RW depiction appears rather similar in FGATIR and FLAWS. We are convinced that they can be used in analogy and are used as such in clinical work. A further limitation is that all tractographic conclusion have been drawn on group level on HCP and not on individual level due to the heterogeneity of the diffusion MRI data for our clinical protocols. Whether the RW coincides with the culmination point of the DRTx on an individual level is subject to future work.

We have used stimulation contacts from effective DBS electrodes in the tremor indication and found a solid correlation on the right (RW and DRTx) but not on the left side. With a lack of prospective information on tremor ratings, reporting of outcome parameters of this mixed cohort, which dominantly includes ET patients, is rather coarse (CGI rating). Moreover our CGI ratings reflect a bilateral outcome state and do not differentiate for sides potentially negatively interfering with our regression analysis. Our group of retrospectively evaluated ET patients (n = 28) is rather small. Nevertheless, CGI outcome was favourable and our contact estimation group around RW and in a way therefore confirm our anatomy driven analysis.

## 6 Conclusion

According to our analysis the rubral wing of Bot, Schuurman (Bot et al., 2022) and the subthalamic hypointensity of Neudorfer (Neudorfer et al., 2022) represent the terminal and horizontal portion of the crossed DRT which in our experience is potentially the most effective tremor reducing stereotactic target region located in the thalamic-subthalamic region. It is necessary to differentiate DRTx from DRTu with respect to RW and it is our understanding that unnecessary stimulation or − even worse − lesioning of the further posterior residing DRTu might lead to a delayed habituation of tremor control and unnecessary ataxia (in DBS and SLS). RW is already used by different groups as a targetable structure in tremor surgery although the mere holistic characterization of the RW with its connectivity and extension is lacking so far. This paper has tried to close this gap and in a way strives for anatomical plausibility of its role as a major structure for tremor reduction. In the future prospective studies targeting the RW/DRTx are necessary to shed further light on the matter and such studies are the focus of our current research (OPTI-ET, DRKS00032400). In the light of this study utilizing volumetric and connectomic approaches, the RW‘s role as a valid and individually visualizable tremor target for DBS and SLS is strengthened. The utilization of FLAWS/FGATIR sequences for delineating RW enables neurosurgeons to employ a geometrically accurate, yet broadly applicable, personalized MRI grey matter sequence for tremor targeting. However, the volumetric interpretation of the structure might not be unequivocal in every individual single case (especially with respect to its anterior-posterior extensions) and care must be taken to readily appreciate the entire structure during the planning process. Reduced individual SNR might result in low quality RW depiction with FLAWS/FGATIR and can consequentially necessitate the use of adjunct imaging modalities (e.g. DWI) to avoid detrimental side effects of tremor surgery.

## CRediT authorship contribution statement

**Volker A. Coenen:** Writing – review & editing, Writing – original draft, Visualization, Supervision, Project administration, Conceptualization. **Alexander Rau:** Writing – review & editing, Visualization, Resources, Data curation. **Horst Urbach:** Writing – review & editing, Project administration. **Bastian E.A. Sajonz:** Writing – review & editing, Resources, Project administration, Data curation. **Marco Reisert:** Writing – review & editing, Writing – original draft, Visualization, Methodology.

## Declaration of competing interest

The authors declare the following financial interests/personal relationships which may be considered as potential competing interests: Unrelated: V.A.C. receives a collaborative grant from BrainLab (Munich, Germany). He is a consultant for Ceregate (Munich, Germany) and Cortec (Freiburg, Germany). He has an ongoing IIT with Boston Scientific (USA) and has received personal honoraria and travel support for lecture work from Boston Scientific (USA). Unrelated: B.E.A.S. received a research grant from Ceregate (Munich, Germany) and received honoraria as a consultant for Precisis, Heidelberg, both unrelated to this work. All other authors declare no conflicts of interest.

## Data Availability

The data that has been used is confidential.
